# Numerical simulation of blasting behavior of rock mass with cavity under high in-situ stress

**DOI:** 10.1038/s41598-024-67088-5

**Published:** 2024-07-11

**Authors:** Hai Rong, Nannan Li, Chen Cao, Yadi Wang, Jincheng Li, Mingda Li

**Affiliations:** College of Mining, Liaoning Technical University, Fuxin, 123000 China

**Keywords:** Numerical simulation, Empty hole effect, High in-situ stress, Circumferential stress, Mineralogy, Petrology

## Abstract

With the shift of coal seam mining to the deep, the in-situ stress of coal and rock mass increases gradually. High ground stress can limit the generation of rock cracks caused by blasting, and blasting usually shows different crushing states than low stress conditions. In order to study the blasting expansion rule of rock mass with cavity under high ground stress and the rock mass fracture state under different side stress coefficients. In this paper, the effective range of blasting and the stress distribution under blasting load are analyzed theoretically. The RHT (Riedel-Hiermaier-Thoma) model is used to numerically simulate the blasting process of rock mass with cavity under different ground stress, and the influence of ground stress and lateral pressure coefficient on the crack growth of rock mass is studied. The results show that when there is no ground stress, the damage cracks in rock mass are more concentrated in the horizontal direction and the fracture development tends to the direction where the holes are located, which confirms the guiding effect and stress concentration effect of the holes in rock mass, which helps to promote the crack penetration between the hole and the hole. The length difference of horizontal and vertical damage cracks in rock mass increases with the increase of horizontal and vertical stress difference. Under the same lateral stress coefficient, the larger the horizontal and vertical stress difference is, the stronger the inhibition effect on crack formation is. For blasting of rock mass with high ground stress, the crack formation length between gun holes decreases with the increase of stress level, and the crack extends preferentially in the direction of higher stress. Therefore, the placement of gun holes along the direction of greater stress and the shortening of hole spacing are conducive to the penetration of cracks between gun holes and empty holes. The research can provide reference for rock breaking behavior of deep rock mass blasting.

## Introduction

Underground rock blasting possesses the advantages of rapidity and high efficiency, finding wide applications in various aspects of underground mining engineering practices, such as coal excavation, rock tunneling, and mitigating dynamic disasters caused by ground pressure in mines. Conventional blasting lacks precision control, leading to phenomena like over-excavation or under-excavation in mining operations. Under-excavation results in poor blasting effects, while over-excavation not only escalates later repair costs but also causes permanent damage to the surrounding rock formations^1–4^. Directional controlled blasting is a technical means to achieve precise control over conventional blasting, which can reduce project costs, enhance operational efficiency, and prevent unnecessary rock damage. There are various methods to achieve directional controlled blasting, among which the technique of borehole directional blasting exhibits advantages such as low cost and simplicity of operation. This method has been applied in engineering practices and has shown promising results^5^. Borehole directional blasting involves introducing additional boreholes between two conventional blast holes to divert the blast-induced cracks towards the direction of the borehole, achieving the objective of directional blasting, termed as the “borehole effect”^6^. Theoretically, stress waves generated by an explosion propagate within the rock. Near the free surface, due to reflection, compression waves transform into tension waves. If the rock's tensile strength near the free surface is lower than the intensity of the tensile stress waves, the rock undergoes tensile failure. The borehole acts as the free surface of the blasting rock. A larger borehole radius results in a larger wavefront area, leading to a larger region of tensile failure in the rock near the free surface^7^.

Compared to surface rock blasting, underground rock formations pose more complex geological conditions, and directly observing the blasting effects is challenging. Therefore, research on borehole directional blasting primarily relies on laboratory experiments or numerical computation methods. Experimental studies primarily utilize polymethyl methacrylate (PMMA) to simulate surrounding rock and investigate the influence of boreholes on stress wave propagation and the development process of blast-induced main cracks under various conditions using pre-drilled holes or grooving techniques^8–13^. Research findings indicate that boreholes distinctly guide the expansion of explosion-induced cracks, with the spacing between blast holes being a critical parameter for the main crack penetrating the borehole.

In terms of numerical computations, Li Xiaohan et al.^14^ used the Riedel-Hiermaier-Thoma (RHT) model to conduct numerical research on the blasting process of single blasting hole, and studied the influence of the magnitude of original stress and lateral pressure coefficient on the fracture zone and crack growth. Yi Changping^15^ obtained by numerical simulation that the crack propagation was controlled by the explosion load near the blasting hole, and the plateau potential stress would affect the crack propagation in the far field. The crack propagates in the direction of high initial pressure. Pu et al.^16^ using the finite difference method, reached similar conclusions. Qian et al.^17^ employed the rock fracture analysis software RFPA-dynamic to calculate the impact of empty hole blasting parameters on crack propagation. Yang et al.^18^ applied LS-DYNA software to conduct three-dimensional numerical simulation of rock damage evolution during deep tunnel excavation, with special emphasis on the combined effects of stress redistribution of surrounding rock mass and blasting damage. The effect of repeated blasting loads on actual millisecond delay blasting damage elongation is studied.

It is worth noting that studies on empty hole blasting generally do not consider in-situ stresses. In other words, research outcomes are solely applicable to surface and shallow rock blasting projects. Deep-seated rock formations possess higher original rock stresses, which significantly influence the extent of crack propagation^15^. In this study, the ANSYS/LS-DYNA software was utilized to establish a dynamic model of dual-hole explosion-induced crack propagation containing empty holes. Various in-situ stress conditions in rock mass were analyzed to understand the evolution process of explosion-induced cracks under different conditions, providing valuable insights for deep-seated rock blasting engineering.

## Methods and materials

### Rock blasting theory

Following the initiation of explosives in coal rock mass, a significant amount of heat energy, explosive gas, and powerful shock waves are generated, sequentially forming distinct zones: the cavity expansion zone, crushing zone, fissure zone, and elastic zone. Within the cavity expansion area, a portion of the explosion's energy is utilized to compress the expanding cavity created by the coal and rock mass. In the crushing area, the majority of the explosion's energy is expended in the extensive crushing of the rock. In the fissure area, the explosive gas primarily exerts its force at the crack tips. When the gas pressure exceeds the fracture strength of the crack tip, the cracks begin to propagate further, and multiple cracks develop simultaneously, intersecting each other. Consequently, the integrity of the rock in this region diminishes, leading to a redistribution of mechanical parameters such as compressive strength and tensile strength. In the elastic region, the outward transmission of stress waves generated by the explosion gradually weakens, and the dynamic tensile strength becomes insufficient to further fracture the rock once it reaches this elastic region^19–22^.

The shock wave pressure after explosive explosion is

1 $$P_{0} = \frac{1}{1 + \gamma }\rho_{0} D_{V}^{2}$$

where *P*_0_ is the explosive pressure; *ρ*_0_ is the density of the explosive; *D*_V_ is the detonation speed of explosive; γ is the adiabatic expansion index of explosive gas.

The pressure of the shock wave load transmitted into the hole wall in the rock is

2 $$P = \frac{{2\rho c_{p} }}{{\rho {\text{c}}_{{\text{p}}} + \rho_{0} D_{V} }}P_{0}$$

where *c*_p_ is the sound velocity of the rock; *ρ* is the density of the rock.

The shock wave enters the rock through transmission, where it gradually decays into stress waves and elastic waves. At the interface between the fracture zone and the elastic zone, the stress state of the rock can be expressed as follows: *σ*_i_ ≥ *σ*_td_, where *σ*_i_ is the stress at a certain point in the rock. *σ*_td_ is the dynamic uniaxial tensile strength of rock.

The radius of the crushing zone is affected by the structure of the charge. When the charge is coupled, the radius of the crushing zone is

3 $$R_{{\text{c}}} = \left( {\frac{{2\rho {\text{c}}_{{\text{p}}} }}{{\rho {\text{c}}_{{\text{p}}} + \rho D_{V} }}\frac{{\rho D_{V}^{2} B}}{{4\sqrt 2 \sigma_{{{\text{cd}}}} }}} \right)^{{\frac{1}{\alpha }}} {\text{r}}_{{\text{b}}}$$

When the charge is coupled, the radius of the fracture zone is

4 $$R_{{\text{p}}} = \left( {\frac{{2\rho {\text{c}}_{{\text{p}}} }}{{\rho {\text{c}}_{{\text{p}}} + \rho D_{V} }}\frac{{\rho D_{V}^{2} B}}{{4\sqrt 2 \sigma_{{{\text{cd}}}} }}} \right)^{{\frac{1}{\alpha }}} {\text{r}}_{{\text{b}}} \left( {\frac{{\sigma_{{{\text{cd}}}} }}{{\sigma_{{{\text{td}}}} }}} \right)^{{\frac{1}{\beta }}}$$

5 $$B = \left[ {\left( {1 + {\text{b}}} \right)^{2} + \left( {1 + {\text{b}}^{2} } \right) - 2\mu_{{\text{d}}} \left( {1 - \mu_{{\text{d}}} } \right)\left( {1 - {\text{b}}} \right)^{2} } \right]^{\frac{1}{2}}$$

where *σ*_cd_ is rock dynamic uniaxial compressive strength; *α*, *β* are the attenuation coefficients of stress waves in the crushing zone and the crack zone, respectively. The diameter of the charge hole; *b* is the lateral pressure coefficient; *μ*_d_ is the dynamic Poisson ratio of rock; The meaning and value range of the parameters in this paper can be referred to^23,24^.

#### Computational method

**Figure 1:**
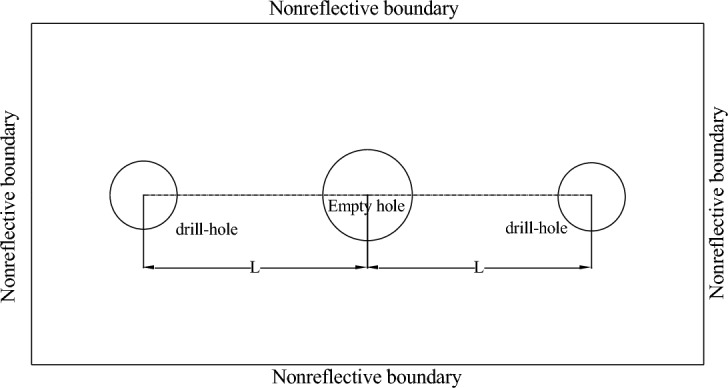
Schematic diagram of numerical model.

The detonation of explosives within the rock induces significant deformation or even fracture of the rock mass. Employing the Arbitrary Lagrange-Eulerian (ALE) algorithm in ANSYS/LS-DYNA software, a cm-g-us unit plane computational model was established, as shown in Fig. 1. The model dimensions are 600 cm × 600 cm. Various quasi-two-dimensional models were constructed with distances of 1 m, 1.25 m, 1.5 m, 2 m, and 2.5 m between the borehole and the empty hole. The borehole diameter is 42 mm, and the empty hole diameter is 100 mm. The computational duration is 1000 us. Symmetrical constraints were applied to nodes on the symmetric plane, while non-reflective boundaries were imposed on all sides. The geometric model includes three materials: rock, explosive, and air, modeled using the Lagrange algorithm for rock and the Euler algorithm for air and explosive materials.

### Materials

#### Rock

Rock material is a typical brittle material and exhibits different properties. Under external forces, these materials develop cracks that intersect, leading to significant macroscopic fractures and material damage. The process of fracture development in rock materials undergoes various stages, including elastic mechanics, fracture mechanics, and damage mechanics^25^.

An et al.^26^ conducted a systematic review of the numerical simulation of rock blasting, showing that the selected material model has a great influence on the results of the numerical simulation. Considering the influence of material model, LS-DYNA provides many models for simulating rock or rock-like materials, such as the continuous surface cap model (CSCM)^27^, the Cowper-Symonds model^28^, the Johnson-Holmquist model^29,30^, the Karagozian-Case model^31,32^, etc. However, parameters for the CSCM model are difficult to determine^33^. The Cowper-Symonds model is too simple to characterize the dynamic response of rocks under blasting loads (LSTC, 2018). The Johnson-Holmquist model is better in simulating the compressive damage of materials while it is unsuitable for modeling the tensile damage^34^. On the contrary, the Karagozian-Case model is good at modeling the tensile damage of materials but it is weak in simulating the compressive damage^35^. Recently, the RHT model has been extensively used to simulate rock or rock-like materials^36,37^. Studies by Wang et al.^38^ have demonstrated that the RHT model exhibits good applicability for simulating rock material behavior under blasting.

**Figure 2:**
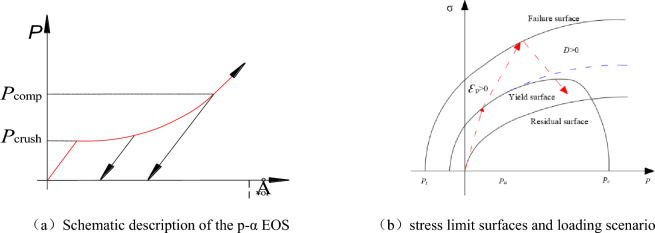
RHT model.

In the RHT model, pressure is expressed using the Mie–Greisen form, with a polynomial Hugoniot curve and a p-α compaction relationship^39,40^. A schematic description of the p-α EOS is shown in Fig. 2a. In a compaction model, when the pressure value is below the pore crush pressure, the model is elastic. Once the pressure exceeds the pore crush pressure, pore collapse reduces the volumetric stiffness of the material, resulting in the reduction of the effective bulk modulus. The relationship between the pressure and volumetric strain is non-linear. When the pressure exceeds the pore crush pressure, unloading occurs along the current elastic stiffness. This results in a permanent volumetric strain at zero pressure. Subsequent reloading occurs along the unloading curve. To characterize pore collapse behavior, an internal variable *α* is used to represent the porosity of the material as the fraction between the matrix material and the porous material. The porosity of *α* decreases with increasing pressure, making the loading irreversible. When the pressure reaches the pore crush pressure and compaction pressure, *α* equals 0 and 1, respectively.

In the RHT model, three stress limit surfaces are used to account for both reductions in strength along different meridians as well as strain rate effects. These limit surfaces include the initial elastic yield surface, and residual friction surface. A typical loading scenario is shown in Fig. 2b, the arrows indicate that the model is elastic until the stress reaches the initial yield surface. Beyond this surface, plastic strain begins to accumulate. The plastic strain and hardening properties of the material are applied to form an effective yield surface, which is created from an interpolation between the initial yield surface and failure surface. As the stress reaches the failure surface, damage from plastic strain accumulates, which causes the failure surface to soften. This results in the formation of the post-failure stress limit surface, which is created by interpolating values between the failure surface and residual friction surface.

The equation of state of the RHT model is as follows:^13^

6 $$p\left( {\rho ,e} \right) = \frac{1}{\alpha }A_{1} h + A_{2} h^{2} + A_{3} h^{3} + \left( {B_{0} + B_{1} h} \right)\rho_{0} e,h > 0$$

7 $$p\left( {\rho ,e} \right) = \frac{1}{\alpha }T_{1} h + T_{2} h^{2} + B_{0} \rho_{0} e,h < 0$$

where *h* is the volume strain, and the expression is $\mu = \rho /\rho_{0} - 1$:$\rho_{0}$ is the initial density of rock; $\rho$ is the density of the material in the compression process; $e$ is the initial internal energy; *A*_*1*_, *A*_*2*_, *A*_*3*_, *B*_*0*_, *B*_*1*_, *T*_*1*_, *T*_*2*_ are state equation parameters.

The failure surface describes the maximum deformation pressure that the material can withstand, and its equation can be expressed as

8 $$\sigma_{f} \left( {p,\theta ,\mathop \varepsilon \limits^{ \cdot } } \right) = f_{c} \cdot \sigma_{TXC} \left( {p_{s} } \right) \cdot \left( {\mathop \varepsilon \limits^{ \cdot } } \right) \cdot R_{3} \left( \theta \right)\lambda$$

where $\sigma_{TXC} \left( {p_{s} } \right)$ quasi-static failure surface compressive meridian equivalent stress intensity; $f_{c}$ is uniaxial compressive strength, MPa; $R_{3} \left( \theta \right)$ is Rodeo Angle factor; $p_{s} = p/F_{r} \left( {\mathop \varepsilon \limits^{ \cdot } } \right)$ quasi-static pressure, $F_{r} \left( {\mathop \varepsilon \limits^{ \cdot } } \right)$ is the dynamic strain rate enhancement factor, which reflects the effect of strain rate on the constitutive relationship of concrete materials, the expression is

9 $$F_{r} \left( {\mathop \varepsilon \limits^{ \cdot } } \right) = \left\{ \begin{gathered} \left( {{{\mathop \varepsilon \limits^{ \cdot } } \mathord{\left/ {\vphantom {{\mathop \varepsilon \limits^{ \cdot } } {\mathop {\varepsilon_{0}^{c} }\limits^{ \cdot } }}} \right. \kern-0pt} {\mathop {\varepsilon_{0}^{c} }\limits^{ \cdot } }}} \right)^{{\beta_{c} }} ,p \ge f_{c} /3 \hfill \\ \frac{{p + f_{t} /3}}{{f_{c} /3 + f_{t} /3}}\left( {{{\mathop \varepsilon \limits^{ \cdot } } \mathord{\left/ {\vphantom {{\mathop \varepsilon \limits^{ \cdot } } {\mathop {\varepsilon_{0}^{c} }\limits^{ \cdot } }}} \right. \kern-0pt} {\mathop {\varepsilon_{0}^{c} }\limits^{ \cdot } }}} \right)^{{\beta_{c} }} + \left( {{{\mathop \varepsilon \limits^{ \cdot } } \mathord{\left/ {\vphantom {{\mathop \varepsilon \limits^{ \cdot } } {\mathop {\varepsilon_{0}^{t} }\limits^{ \cdot } }}} \right. \kern-0pt} {\mathop {\varepsilon_{0}^{t} }\limits^{ \cdot } }}} \right)^{{\beta_{t} }} ,p \le - f_{t} /3 \hfill \\ \frac{{p - f_{c} /3}}{{ - f_{c} /3 - f_{c} /3}}\left( {{{\mathop \varepsilon \limits^{ \cdot } } \mathord{\left/ {\vphantom {{\mathop \varepsilon \limits^{ \cdot } } {\mathop {\varepsilon_{0}^{t} }\limits^{ \cdot } }}} \right. \kern-0pt} {\mathop {\varepsilon_{0}^{t} }\limits^{ \cdot } }}} \right)^{{\beta_{t} }} , - f_{t} /3 < p < f_{t} /3 \hfill \\ \end{gathered} \right.$$

where $f_{t}$ is the uniaxial tensile strength, MPa; $\mathop {\varepsilon_{0}^{c} }\limits^{ \cdot }$、$\mathop {\varepsilon_{0}^{t} }\limits^{ \cdot }$、$\mathop \varepsilon \limits^{ \cdot }$ is the strain rate; $\beta_{c}$ is compressive strain rate coefficient; $\beta_{t}$ is the tensile strain rate coefficient.

In the RHT model, the damage evolution equation is as follows:

10 $$0 \le D = \sum {\frac{{\Delta \varepsilon_{p} }}{{\varepsilon_{p}^{f} }}} \le 1$$

11 $$\varepsilon_{p}^{f} = \left[ {p^{ * } - \left( {1 - D} \right)p_{t}^{ * } } \right]^{{D_{2} }} \ge \varepsilon_{f,\min }$$

where *D* is the damage variable; $\cdot \varepsilon_{p}$ is cumulative plastic deformation; $\varepsilon_{p}^{f}$ is the failure plastic strain; $p^{ * }$ is the standardized failure pressure; $\varepsilon_{f,\min }$ is the minimum allowable plastic strain.* D*_*1*_ and *D*_*2*_ are constants. The damage variable *D* is between 0 and 1, and the greater *D* is, the greater the damage degree of the material. The parameters of the RHT model adopted in this paper are shown in Table 1. Parameters are taken from Xiaohan Li’s paper ^14^.

**Table 1:** RHT parameters of rocks.

Parameter	Value	Parameter	Value
Mass density (kg/m^3^)	2660	Break compressive strain rate	3E + 25
Elastic shear modulus (GPa)	21.9	Break tensile strain rate	3E + 25
Relative shear strength	0.18	Lode angle dependence factor Q_0_	0.68
Relative tensile strength	0.04	Lode angle dependence factor B	0.01
Parameter for polynomial EOS T_1_ (GPa)	35.27	Compressive yield surface parameter	0.53
Parameter for polynomial EOS T_2_ (GPa)	0	Tensile yield surface parameter	0.7
Damage parameter D_1_	0.04	Crush pressure (MPa)	125
Damage parameter D_2_	1.0	Compaction pressure (GPa)	6
Hugoniot polynomial coefficient A_1_ (GPa)	35.27	Shear modulus reduction factor	0.5
Hugoniot polynomial coefficient A_2_ (GPa)	39.58	Eroding plastic strain	2.0
Hugoniot polynomial coefficient A_3_ (GPa)	9.04	Minimum damaged residual	0.01
Failure surface parameter A	1.60	Porosity exponent	3.0
Failure surface parameter N	0.61	Initial porosity	1.0
Residual surface parameter AF	1.60	Pressure influence on plastic flow in tension	0.001
Residual surface parameter NF	0.61	Tensile strain rate dependence exponent	0.036
Parameter for polynomial EOS B_0_	1.22	Compressive strength (MPa)	167.8
Parameter for polynomial EOS B_1_	1.22	Compressive strain rate dependence exponent	0.032
Reference compressive strain rate	3E − 05	Gruneisen gamma	0
Reference tensile strain rate	3E − 06		

#### Explosive parameters and equation of state

The high performance explosive material model *MAT_HIGH_EXPLOSIVE_BURN built in ANSYS/LS-DYNA software and the JWL equation of state are used to describe the volume, pressure and energy characteristics of the explosive products in the explosion process. The expression is as follows:

$$P = A\left( {1 - \frac{\omega }{{R_{1} V}}} \right)\exp \left( { - R_{1} V} \right) + B\left( {1 - \frac{\omega }{{R_{2} V}}} \right)\exp \left( { - R_{2} V} \right) + \frac{{\omega E_{0} }}{V}$$

where *P* is the detonation pressure; *V* is the relative volume; *E*_0_ is the initial specific internal energy; *A*, *B*, *R*_*1*_, *R*_*2*_, *ω* are material constants, as shown in Table 2.

**Table 2:** Parameters for the explosive material and JWL EOS^41^.

ρ_e_/(kg/m^3^)	*V*_0_*D*(m/s)	P_CJ_/GPa	A/GPa	B/GPa	R1	R2	ω	E_0_/GPa
1320	6690	16	586	21.6	5.81	1.77	0.282	7.38

#### Air material

Air adopts the empty matter material model *MAT_NULL, and its equation of state is expressed in linear polynomial *EOS_LINER_POLYNOMIAL:

$$P = C_{0} + C_{1} V + C_{2} V^{2} + C_{3} V^{3} + \left( {C_{4} + C_{5} V + C_{6} V^{2} } \right)E_{0}$$

In the formula, *C*_0_ ~ *C*_6_ are the relevant parameters of the equation. Among them, *C*_4_ = *C*_5_ = 0.4, *E*_0_ = 2500 MJ/m^3^, *V* = 1.0, and other parameters are 0.

## Results and discussion

### Rock crack formation process

#### Without in-situ stress

**Figure 3:**
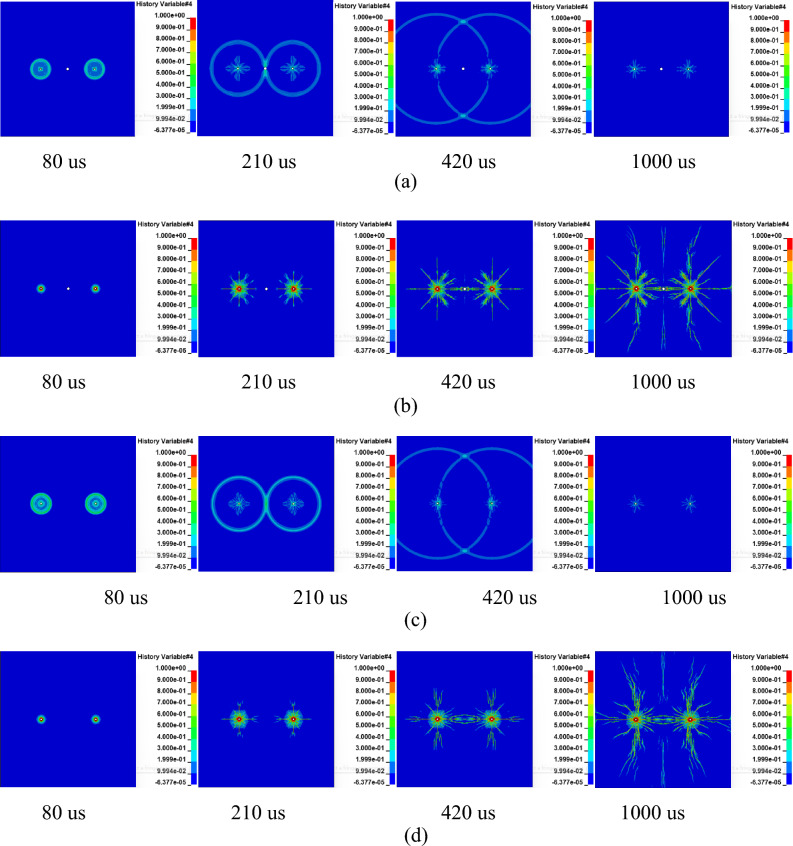
Stress cloud map and rock damage under no in-situ stress when L = 200 cm. (**a**, **b** represent stress contour maps and crack formation with empty holes; **c**, **d** represent stress contour maps and crack formation without empty holes).

In the absence of applied in-situ stress, Fig. 3a–d illustrate the process of rock blasting-induced crack formation and stress contour maps for rock with and without empty holes at a borehole spacing of 200 cm.

The results depict that upon detonation, the shockwave contacting the borehole wall causes rock fragmentation, forming a crushed zone around the borehole, gradually extending to form a fractured region in the surrounding rock. At a distance from the borehole, the shockwave transforms into a stress wave, radiating outward in a circular manner by t = 80 us, forming a fractured region. By t = 210 us, the radial cracks continue to expand as the wave fronts of two stress waves meet, resulting in tensile stress in the tangential direction of the wave front. From the damage cloud map, it can be observed that crack development tends to orient towards the direction of the empty hole, highlighting the guiding effect of the empty hole. At t = 420 us, as stress waves propagate and diminish in intensity, the stress wave reaching the empty hole reflects, causing tensile stress that further leads to rock elongation failure, ultimately resulting in crack penetration between the two boreholes. By t = 1000 us, crack formation ceases.

Comparing crack expansion with and without empty holes, it is evident that fewer but wider cracks appear around the empty holes. During the propagation of stress waves, when the stress wave surpasses the dynamic tensile strength of the rock, radial cracks form until they penetrate or stop at the borehole.

**Figure 4:**
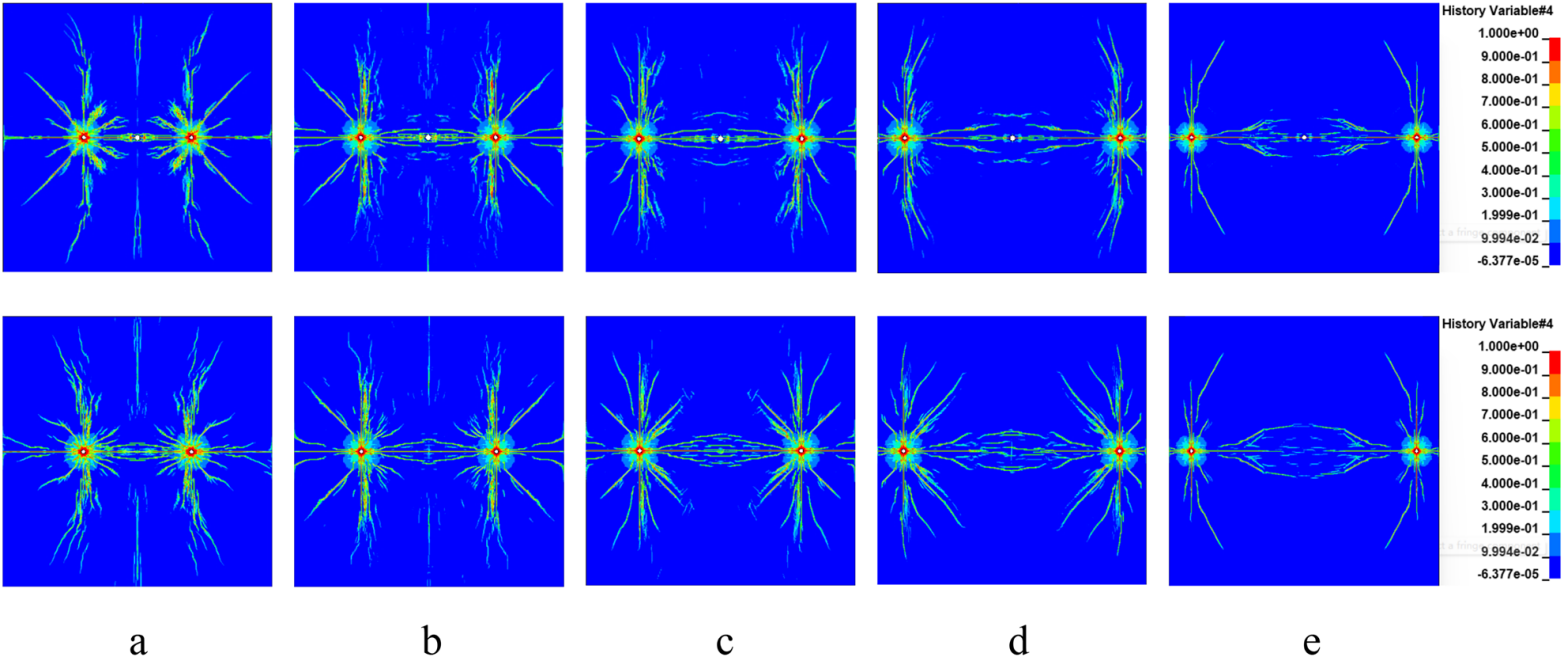
Rock fracture characteristics with different hole spacing (control with or without holes).

Figure 4 illustrates the results of rock explosion-induced crack expansion for coupling charges with borehole diameter of 42 mm and empty hole diameter of 100 mm at different borehole spacings of 2 m, 2.5 m, 3 m, 4 m, and 5 m under conditions with and without empty holes.

From Fig. 4, it can be observed that as the borehole distance gradually increases from 2 to 5 m, the superposition effect of stress waves weakens, resulting in a reduction in the number of damage-induced cracks between boreholes and a decrease in the empty hole effect. For distances between L = 2–4 m, radial penetrating cracks form between boreholes regardless of the presence of empty holes. In scenarios without empty holes, there are more dispersed crack developments between boreholes. However, at a borehole spacing of 5 m without empty holes, cracks around the boreholes are distant and fail to form penetrating cracks. Conversely, with empty holes, crack formation between boreholes tends to converge towards the empty hole. There are fewer concentrated cracks, and they are more centralized, nearly penetrating between boreholes, demonstrating the prominent guiding effect of the empty hole.

**Figure 5:**
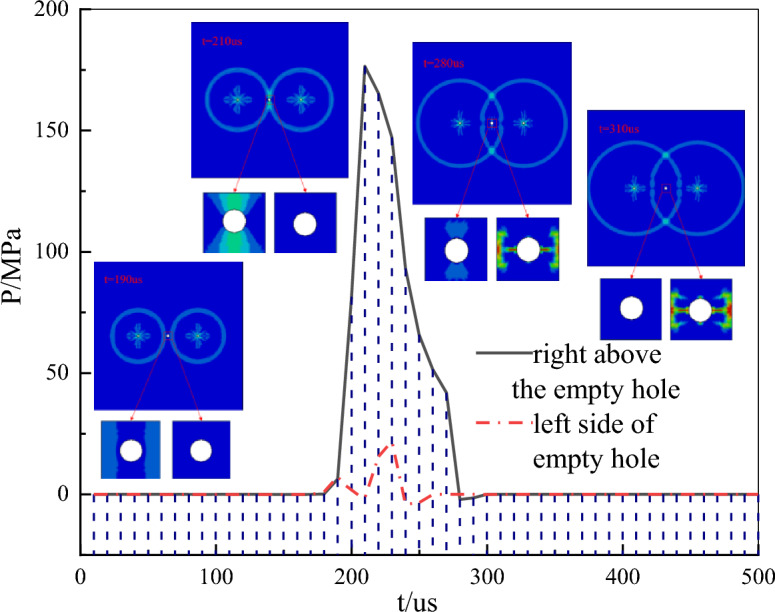
Evolution of pore pressure when L = 200 cm.

Considering the symmetry of the model, monitoring units were placed directly above and to the left of the empty hole. As shown in Fig. 5, the black and red curves represent the pressure curves directly above and to the left of the empty hole, respectively. Surrounding the curves are pressure contour maps and localized magnifications of the damage around the empty hole.

The results indicate that following the explosion, compressive stress waves propagate toward the empty hole, compressing the medium on the left and right sides of the empty hole and inducing shear deformation in the upper and lower mediums. At t = 190 us, the compressive wave reaches the empty hole, where the pressure at the monitoring unit is relatively small and does not cause damage to the empty hole. By t = 210 us, stress above the empty hole reaches its maximum value at 176.49 MPa, resulting in shear failure above the empty hole. At t = 280 us, the shear failure area enlarges, with tensile cracks appearing on both sides, indicating a failure pattern around the empty hole involving shear failure followed by tensile failure. At t = 230 us, the pressure on the left side of the empty hole reaches a maximum of 21.96 MPa. At t = 240 us, the maximum tensile stress is 4.03 MPa. By t = 310 us, tensile cracks appear above and below the empty hole as well as on both sides, with more prominent tensile cracks on the sides continuing to expand until they penetrate the main crack on the left side.

### In-situ stress conditions

Considering that boreholes exist within a rock mass subjected to in-situ stress conditions^42–44^, the model is subjected to horizontal σ_x_ and vertical σ_y_ in-situ stress to align with real conditions. To investigate the effects of varying in-situ stress magnitudes and principal stress directions on the rock blasting-induced fracture process, this study examines crack formation under three scenarios: static in-situ stress conditions and non-static in-situ stress conditions with ten different in-situ stress configurations, as presented in Table 3.

**Table 3:** In-situ stress loading conditions.

Field of stress	Working condition	$\sigma$_x_/MPa	$\sigma$_y_/MPa	$\lambda$** = **$\sigma$_x_/$\sigma$_y_
Hydrostatic in-situ stress field	1	10	10	1
	2	20	20	1
	3	30	30	1
	4	40	40	1
	5	50	50	1
Non-hydrostatic in-situ stress field	6	20	10	2
	7	60	30	2
	8	30	10	3
	9	60	20	3
	10	80	20	4

#### Hydrostatic in-situ stress field

**Figure 6:**
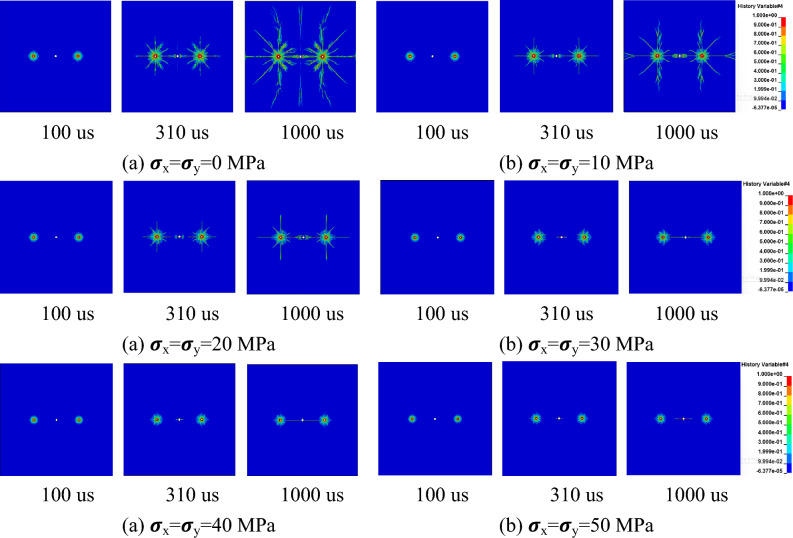
Crack formation between gun holes with a spacing of 2 m.

**Figure 7:**
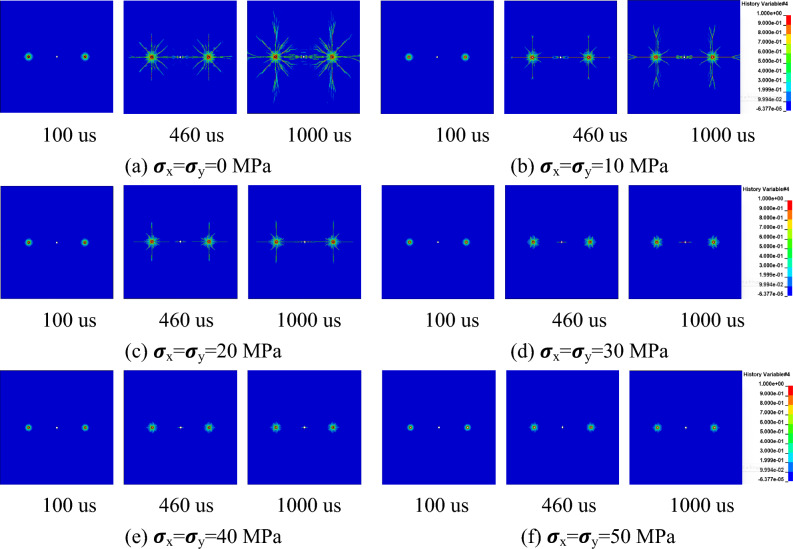
Crack formation between gun holes with a spacing of 3 m.

Under different hydrostatic stress field conditions, the crack formation process after detonation is shown in Figs. 6 and 7 with the shothole spacing of 2 m and 3 m.

The results indicate that within 100us, the speed and direction of crack formation are essentially consistent under different in-situ stress conditions, indicating minimal influence of in-situ stress on the blasting-induced fracture process. This is attributed to the significantly higher explosive load at this stage compared to in-situ stress^45^. At 310 us and 460 us, cracks between boreholes and empty holes are fully penetrated under no in-situ stress conditions, whereas at the same instants, cracks between boreholes and empty holes do not penetrate under in-situ stress conditions. Specifically, at a 2 m spacing between boreholes, static in-situ stress below 40 MPa allows for complete penetration of cracks between boreholes and empty holes; at a 3 m spacing, static in-situ stress needs to be below 20 MPa for penetration to occur. This suggests an inhibitory effect of in-situ stress on rock blasting-induced fracture.

#### Non-hydrostatic in-situ stress field

**Figure 8:**
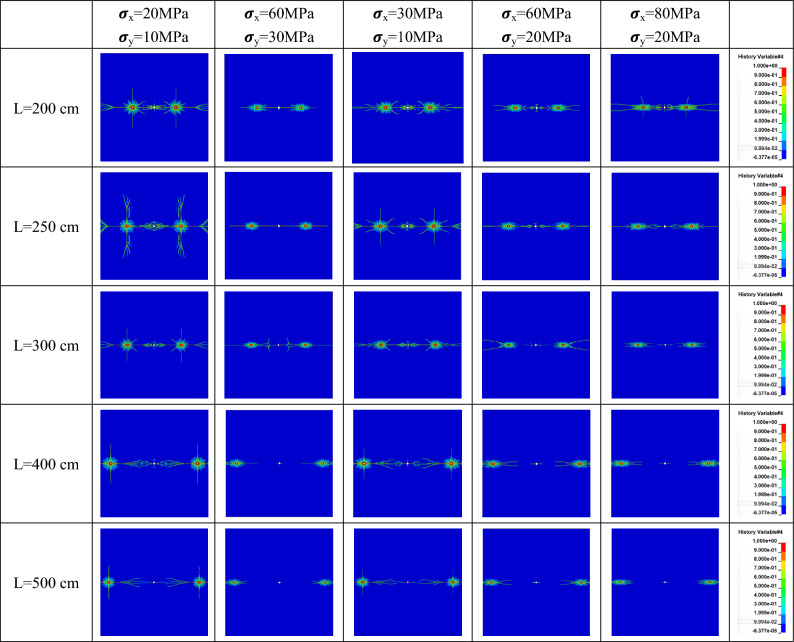
Rock crack growth under different spacing and different non-hydrostatic geostress.

Under non-hydrostatic in-situ stress conditions in the horizontal plane (λ = 2, 3, 4), crack formation after detonation with different explosive distances is depicted in Fig. 8.

In contrast to hydrostatic in-situ stress, crack formation under non-hydrostatic in-situ stress conditions displays directional characteristics. The range of rock fractures appears elliptical, with the major axis of the ellipse roughly parallel to the direction of maximum principal stress. This occurs because the circumferential compressive stress along the minor principal stress direction exceeds that along the major principal stress direction, resulting in greater inhibition of crack expansion along the minor principal stress direction.

At the same explosive distance (e.g. L = 200 cm) but under different in-situ stress conditions, as the lateral pressure coefficient increases, the directional characteristics of crack expansion become more pronounced, and the inhibitory effect on crack expansion along the minor principal stress direction intensifies. Under similar lateral pressure coefficients but varying stress values, for instance, σ_x_ = 2σ_y_ = 20 MPa versus σ_x_ = 2σ_y_ = 60 MPa, although having the same λ value, the degree of crack expansion inhibition differs. Cracks face more challenges in expansion when there is a substantial difference in in-situ stress values.

The directionality of crack expansion and the degree of inhibition are not only related to the lateral pressure coefficient but also associated with the magnitude of the difference in horizontal and vertical in-situ stress values. Under σ_x_ = 2σ_y_ = 20 MPa and σ_x_ = 3σ_y_ = 30 MPa in-situ stress conditions, cracks between different borehole distances can completely penetrate, forming new free surfaces. Under σ_x_ = 2σ_y_ = 60 MPa, σ_x_ = 3σ_y_ = 60 MPa conditions, complete penetration occurs at distances below 300 cm. At σ_x_ = 4σ_y_ = 80 MPa conditions, complete penetration occurs at distances below 250 cm. Thus, for high in-situ stress rock blasting, when feasible in field conditions, it is advisable to arrange boreholes along the direction of maximum principal stress and adjust borehole distances to achieve optimal crack penetration and rock fracturing effects.

### Evolution of stress field around empty hole

To further elucidate the rock fracturing mechanics in high in-situ stress porous blasting, this section analyzes the dynamic evolution of the surrounding rock stress field during the blasting process.

**Figure 9:**
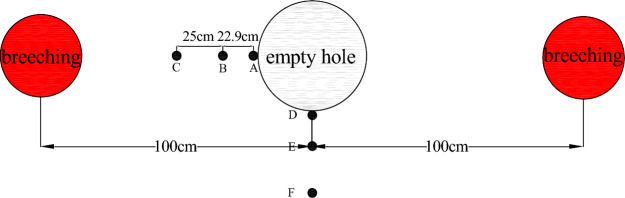
Arrangement of stress observation points L = 200 cm.

Taking the model with a borehole distance of 200 cm as an example, six measuring points (A, D for the empty hole wall, C at the midpoint between the borehole and empty hole, B, E at 25 cm from the empty hole center, and F at 50 cm from the empty hole center) are selected, as illustrated in Fig. 9.

#### No in-situ stress

**Figure 10:**
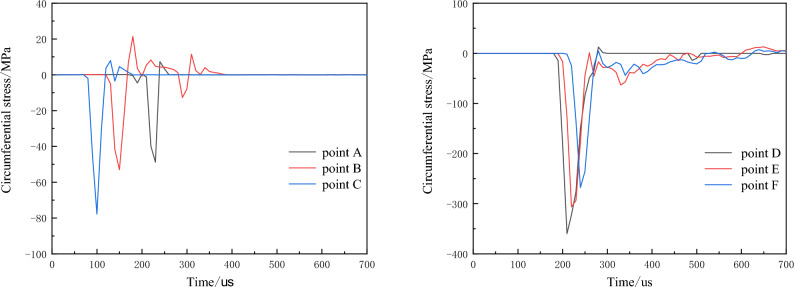
Change curve of circumferential stress with empty hole (σ_x_ = σ_y_ = 0 MPa).

**Figure 11:**
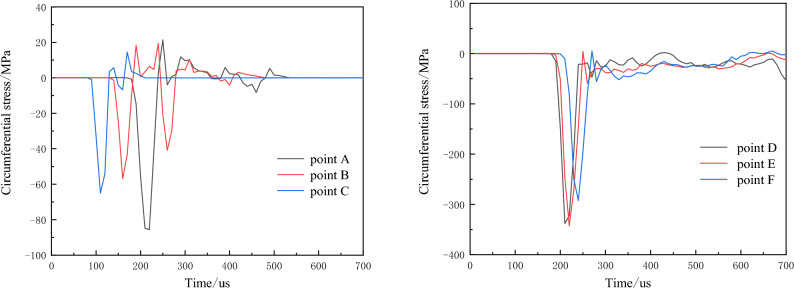
Change curve of circumferential stress without empty hole (σ_x_ = σ_y_ = 0 MPa).

As the tensile strength of the rock mass is significantly lower than its compressive strength, rock fracturing around the empty hole under explosive loading primarily involves radial cracking induced by circumferential tensile stress. Hence, the dynamic variation of circumferential stress is primarily analyzed. Figures 10 and 11 present the circumferential stress-time curves for monitoring points during blasting under no in-situ stress conditions, with negative values representing compression and positive values representing tension (similar below).

Figures 10, 11 illustrate that the circumferential stress around the rock mass is initially compressive during the initial phase of explosive loading. Subsequently, due to radial compression of the rock mass, it shifts towards tensile stress. Notably, points A, B, C, and D, E, F exhibit significant differences in compressive stress.

Upon referencing Table 4, it is observed that monitoring units experience primarily compressive failure without the empty hole. However, with the presence of an empty hole, the stress values are insufficient to cause rock mass failure; instead, tensile stress surpasses the rock's tensile strength, leading to tensile failure. Horizontal tensile stress surpasses vertical tensile stress, while vertical compressive stress greatly exceeds horizontal compressive stress. This suggests that stress waves between boreholes facilitate crack formation, with horizontal direction primarily undergoing tensile failure, while vertical direction primarily experiences compressive failure.

**Table 4:** Stress peak when monitoring the unit with or without empty holes.

	A/MPa	B/MPa	C/MPa	D/MPa	E/MPa	F/MPa
no empty hole tensile stress	21.4	19.4	14.5	1.98	4.40	5.09
no empty hole tensile stress	85.6	56.9	65.0	338.0	342.6	292.2
empty hole tensile stress	7.35	14.8	7.95	12.5	8.64	7.27
empty hole tensile stress	48.8	55.4	77.8	360.0	319.0	267.8

#### Hydrostatic in-situ stress

**Figure 12:**
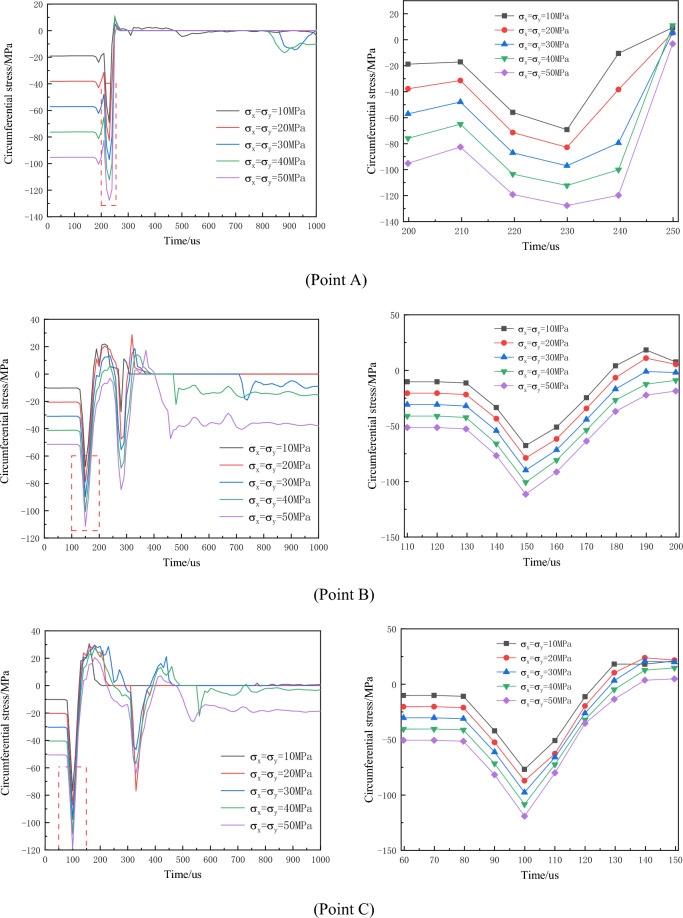

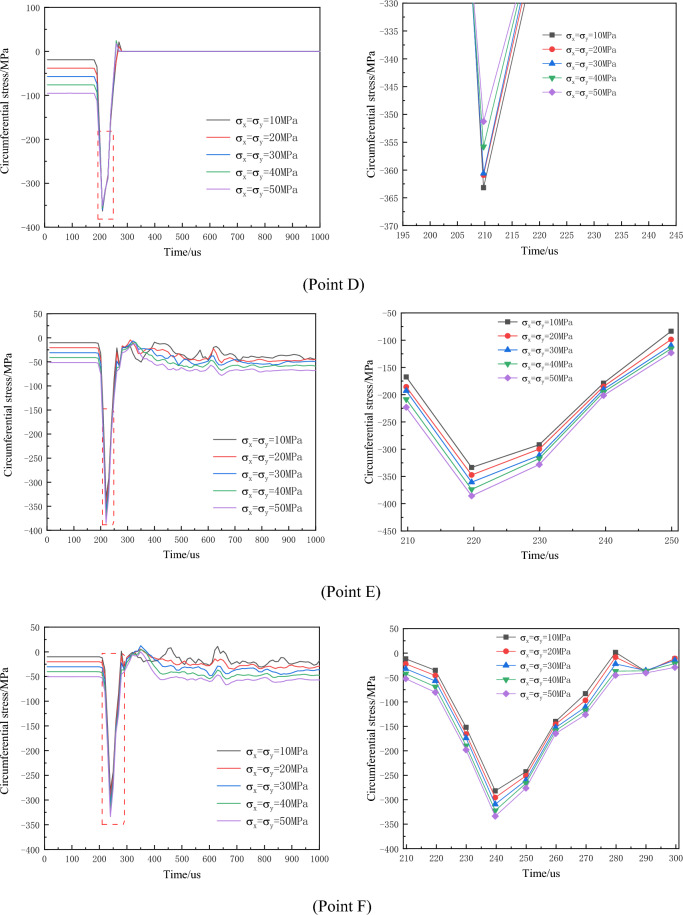
Time history curve of circumferential stress around empty hole under different hydrostatic geostress conditions (local amplification on the right).

Under different levels of hydrostatic in-situ stress, the circumferential stress-time curves at measuring points after the borehole detonation are depicted in Fig. 12.

The results indicate that with increasing in-situ stress, the circumferential tensile stress at the measuring points gradually decreases, and the duration of tensile stress also diminishes. Additionally, the measuring points closer to the empty hole experience greater compressive stress. Under hydrostatic in-situ stress conditions not exceeding 40 MPa, both points B and C register circumferential tensile stress peaks higher than the rock's dynamic tensile strength, leading to crack penetration between the borehole and empty hole. However, at a hydrostatic stress of 50 MPa, point C's circumferential tensile stress peak falls below the rock's dynamic tensile strength, impeding crack penetration between the borehole and empty hole. Consequently, as in-situ stress increases, the circumferential tensile stress between boreholes decreases, exerting an inhibitory effect on the circumferential tensile effect of the explosive load due to in-situ stress.

Due to the influence of the empty hole effect, the initial compressive stress experienced at points A and D on the empty hole wall is approximately twice the applied in-situ stress, and the duration of compressive stress is shorter compared to other measuring points. The magnified partial data in the second column of Fig. 12 indicates that under hydrostatic in-situ stress, the circumferential compressive stress increases with the rise in initial stress level.

#### Non-hydrostatic in-situ stress

**Figure 13:**
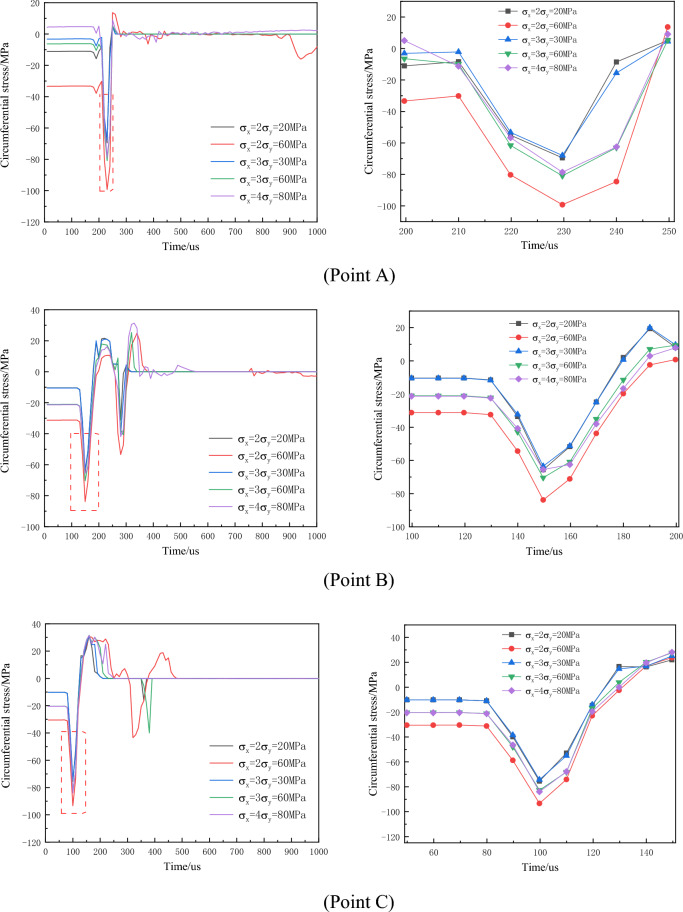

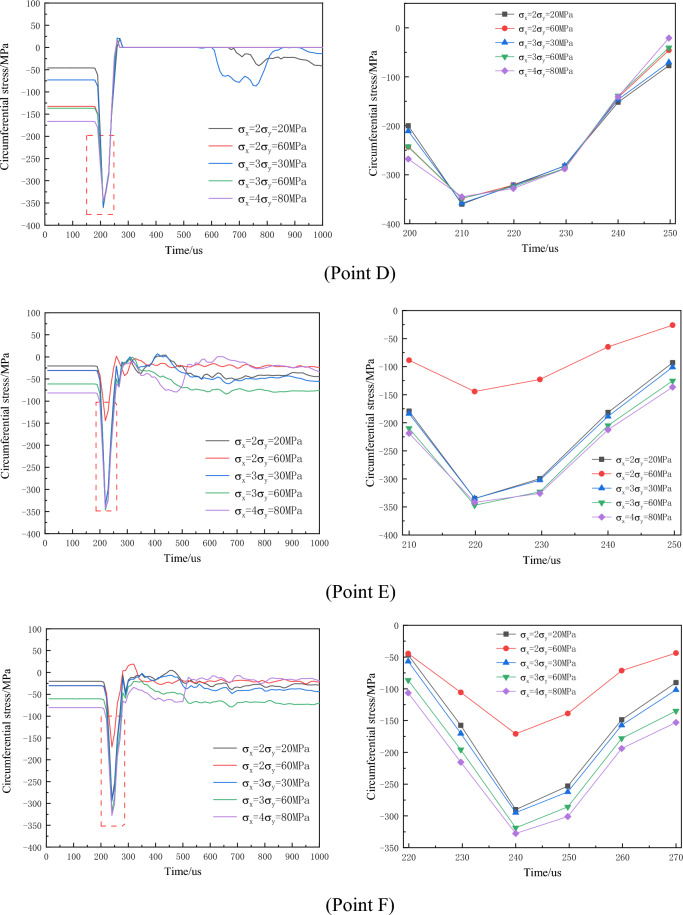
Time history curve of circumferential stress around the empty hole under different non-hydrostatic geostress conditions (local amplification on the right).

The circumferential stress variation-time curves at monitoring points under different non-hydrostatic in-situ stress levels are depicted in Fig. 13.

Primarily, circumferential compressive stress inhibits crack formation, and this inhibitory effect intensifies with increased in-situ stress. Under varying confining pressure coefficients, the influence of in-situ stress on circumferential stress differs in horizontal and vertical directions. In the horizontal direction, the greater the difference in in-situ stress between the horizontal and vertical directions for the same confining pressure coefficient, the higher the circumferential stress, resulting in a stronger inhibition of crack formation. In the vertical direction, as the confining pressure coefficient increases, the circumferential compressive stress amplifies. With the same confining pressure coefficient, larger differences between horizontal and vertical directions result in lower circumferential stress. This analysis indicates that greater in-situ stress exerts a stronger restraining effect on crack formation, causing cracks to predominantly extend towards directions with higher stress.

### Engineering application

**Figure 14:**
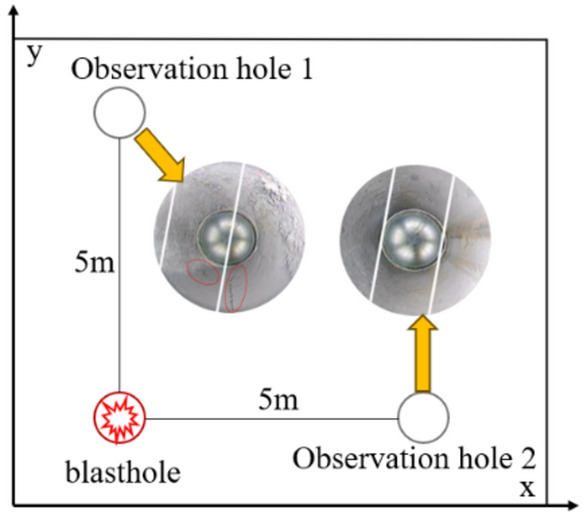
Crack formation peep view.

The in-situ stress measurement results of Tiandi Technology in Geng Cun Coal mine in Henan Province, China show that the maximum horizontal principal stress value of Geng Cun Coal mine is 13.83 MPa, the direction is N36°E, and the minimum principal stress value is 7.29 MPa. According to the above in-situ stress measurement results, the stress values are converted by depth and projected into the X and Y directions of the model respectively. It is determined that 6.42 MPa is applied in the X direction of the model, 13.69 MPa is applied in the Y direction, and the side stress coefficient Y/X is about 2.1. Drill observation holes in the horizontal and vertical directions of the gun holes respectively to observe the crack formation effect of the explosive after detonation, as shown in the Fig. 14. As can be seen from the Fig. 14, under the same distance condition, longitudinal cracks appear in observation hole 1 in the direction of greater stress, while cracks do not appear in observation hole 2 in the direction of lower stress, which is basically consistent with the simulation results, effectively indicating that under the condition of high ground stress, cracks generated by rock blasting preferentially expand to the side with greater stress.

## Conclusion

High ground stress significantly affects the blasting behavior of deep rock mass. The effective range of rock blasting and the stress distribution under explosion load are analyzed theoretically. The blasting behavior under different conditions of high ground stress is numerically simulated by LS-DYNA software. The following conclusions can be drawn:

1. Under high ground stress conditions, compressive stress can be increased and tensile stress reduced. Under the condition of hydrostatic geostress, the circumferential compressive stress increases with the increase of in-situ stress. Under the condition of anisotropic high ground stress, the circumferential compressive stress along the low stress direction is greater than that along the high stress direction, the tensile stress perpendicular to the high stress direction is inhibited, and the crack formation along the low stress direction is inhibited.
2. High ground stress will affect both the fracture zone and crack formation of rock mass. With the increase of ground stress, the total failure of rock mass decreases gradually. Under the condition of equiaxial pressure, the shape of the rock mass fracture zone is circular, and its diameter decreases with the increase of ground stress. Under the condition of anisotropic pressure, the shape of the fracture zone is elliptical, and its long axis is along the higher stress direction.
3. As the difference of ground stress in anisotropy increases, the difference of crack length between the two axes in the fracture zone also increases. Under the same lateral stress coefficient, the larger the stress difference between horizontal and vertical directions is, the stronger the inhibition effect on crack formation is. The crack caused by blasting preferentially expands in the direction of higher stress.
4. In engineering practice, the lateral stress coefficient Y/X is about 2.1, and the crack formation rule is basically consistent with the simulation results, which effectively indicates that under the condition of high ground stress, the cracks generated by rock blasting preferentially expand in the direction of greater stress.

## Data Availability

The datasets used and/or analysed during the current study available from the correspondingauthor on reasonable request. All data generated or analysed during this study are included in this published article.
